# Hallux Valgus Plantar Pressure Distribution before and after a Distal Metatarsal Osteotomy

**DOI:** 10.3390/jcm13061731

**Published:** 2024-03-17

**Authors:** Antonio Mazzotti, Alberto Arceri, Elena Artioli, Laura Langone, Simone Ottavio Zielli, Beatrice Martini, Francesco Traina, Cesare Faldini, Lorenzo Brognara

**Affiliations:** 1 1st Orthopaedics and Traumatologic Clinic, IRCCS Istituto Ortopedico Rizzoli, 40136 Bologna, Italy; antonio.mazzotti@ior.it (A.M.); alberto.arceri@ior.it (A.A.); elena.artioli@ior.it (E.A.); laura.langone@ior.it (L.L.); simoneottavio.zielli@ior.it (S.O.Z.); cesare.faldini@ior.it (C.F.); 2 Department of Biomedical and Neuromotor Sciences (DIBINEM), Alma Mater Studiorum University of Bologna, 40123 Bologna, Italy; beatrice.martini3@studio.unibo.it (B.M.); francesco.traina@ior.it (F.T.); 3 Ortopedia-Traumatologia e Chirurgia Protesica e dei Reimpianti d’anca e di Ginocchio, IRCCS Istituto, Ortopedico Rizzoli, 40125 Bologna, Italy

**Keywords:** hallux valgus, pedobarographic, insole, big toe, osteotomy, peak pressure

## Abstract

**Background:** Hallux valgus (HV) morphological alterations impact forefoot kinetics. Surgery aims to restore both the morphology and function. Plantar pressure (PP) distribution systems represent an innovative additional tool to evaluate the hallux functional outcome after surgery in order to assess the hallux dorsiflexion, coupled with plantar flexion of the first ray. However, the literature reports limited evidence regarding the rebalancing of the plantar pressure distribution following surgery. The purpose of the present study was to examine the PP distribution in HV patients before and after a distal metatarsal osteotomy using a novel anatomically based protocol for in-shoe plantar load analysis during gait. **Methods:** A consecutive series of 18 patients with mild-to-moderate symptomatic HV who underwent a distal metatarsal osteotomy (S.E.R.I. technique) were prospectively evaluated using clinical scores (AOFAS and NRS), radiographic parameters (hallux valgus angle, intermetatarsal angle), and PP measurements via W-INSHOE© (Medicapteurs, Balma, France). Data were collected preoperatively and 12 months after surgery. **Results:** At 12 months follow-up, 3 patients were lost to follow-up, leaving 15 patients (24 HV) for examination. Both clinical and radiographical outcomes showed significant improvements from the pre- to postoperative periods. The PP distribution pattern revealed a significant increase in the peak pressure under the first metatarsal head associated with a significant increase in the peak pressure under the central metatarsals area between the pre- and postoperative periods. **Conclusions:** PP measurement systems hold promise as an additional clinical tool, yet current findings remain inconclusive. Further long-term follow-up studies that incorporate additional parameters are warranted.

## 1. Introduction

Although the biomechanics of the foot are highly complex, many authors agree on the simplification that the first ray bears the highest load during gait, especially in the second half of the stance phase. For physiological gait patterns, the weight distribution should ideally be balanced; even minor deformities can lead to significant alterations in the load distribution [1,2].

Hallux valgus (HV) is a common forefoot deformity that affects both the morphology and function of the foot [3,4]. Morphological changes include a lateral deviation of the hallux and medial displacement of the first metatarsal bone, resulting in progressive subluxation of the first metatarsophalangeal joint [5]. These morphological alterations influence the forefoot kinetics, with HV dysfunction manifesting as reduced loading under the first ray and increased hypermobility. This condition results in a lower pressure under the first metatarsal head and an increase in peak pressure under the central metatarsals, causing central metatarsalgia, mainly at the second and third metatarsal heads [6,7].

Surgery aims to rebalance morphology and function [5]. Numerous surgical techniques have been described. Distal metatarsal osteotomies are widespread in clinical practice due to the procedure’s inherent simplicity and rapid execution [5,8].

Radiographic parameters and clinical scores were predominantly employed to evaluate the extent of postoperative morphological correction and functional outcomes after surgery. However, objective functional evaluations are not commonly mentioned in the literature. Plantar pressure (PP) distribution assessment systems represent an innovative tool used to assess the hallux dorsiflexion coupled with plantar flexion of the first ray, and therefore, the re-establishment of a correct distribution of plantar pressures during walking that could theoretically contribute to objectifying the functional results [9,10,11,12,13,14,15,16,17,18].

The purpose of the present study was to examine the plantar pressure distribution in HV patients before and after a distal metatarsal osteotomy.

## 2. Materials and Methods

After approval from our institutional review board and obtaining written informed consent provided by all patients, a series of consecutive patients with mild-to-moderate symptomatic HV, who underwent a distal metatarsal osteotomy (S.E.R.I. technique) from February 2022 to April 2022, were prospectively evaluated at a single orthopedic university hospital, with a follow up of 12 months.

### 2.1. Patient Selection Criteria

All patients with a diagnosis of mild-to-moderate symptomatic HV were included. In particular, we considered the hallux valgus angle (HVA) and the intermetatarsal angle (IMA), with cut-offs of HVA < 40 and/or IMA < 16.

The exclusion criteria were as follows:

- Patients under 18 years old;
- Rheumatoid arthritis or other inflammatory diseases;
- Neurological disorders or other lower limb disruptions;
- Symptomatic abnormal metatarsal formula with relatively longer second or third metatarsals;
- Prior hallux surgery;
- First metatarsophalangeal joint osteoarthritis that was more than grade 2 according to the Regnauld classification [19].

All confounding factors that potentially lead to disrupting the plantar pressure distribution were excluded. All rheumatic and neuromuscular pathologies, as well as prior hallux surgery, that could potentially lead to altered gait and, consequently, affect the plantar pressure distribution were excluded. An abnormal metatarsal formula can alter the pressure distribution under the central metatarsal area, and arthritis significantly reduces the mobility at the metatarsophalangeal joint, thereby affecting the plantar pressure distribution [6,7].

### 2.2. S.E.R.I. Operative Technique and Postoperative Management

The surgical indication considered the potential application of a distal metatarsal osteotomy, considering the pathological anatomy of the selected HV cases. Among distal osteotomies, the S.E.R.I. technique was the surgeon’s preference. All forefoot surgeries were performed by two experienced foot and ankle orthopedic surgeons.

**Figure 1:**
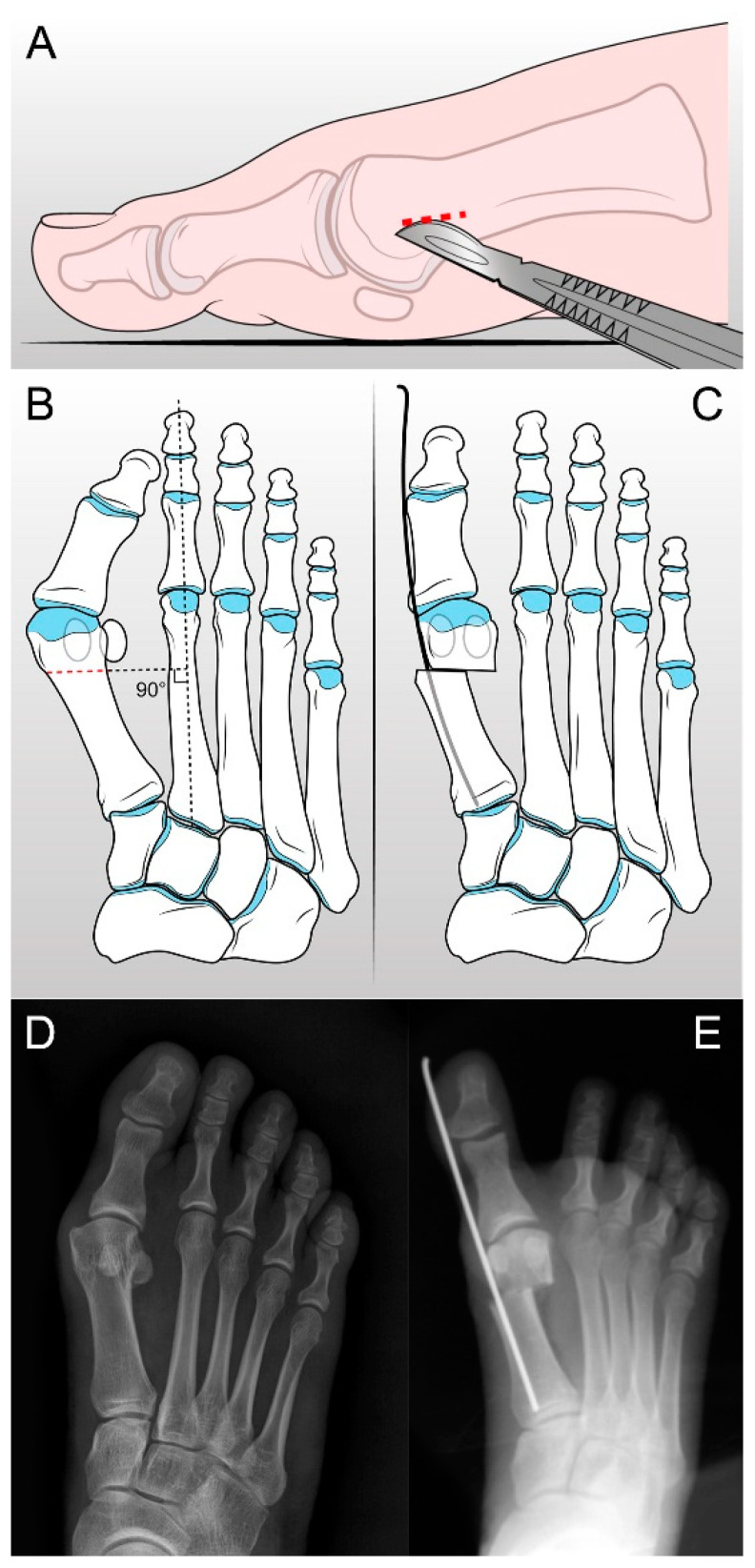
Schematic illustration of distal metatarsal osteotomy according to S.E.R.I. technique.

The patient was placed in a supine position under regional or block anesthesia and tourniquet control. The first surgery step represented the adductor hallucis release by forcing the hallux into a varus position with manual stretching or by performing a tenotomy using a scalpel through a percutaneous approach. The metatarsal neck was exposed through a 0.8 cm incision just proximal to the medial eminence (Figure 1A). The osteotomy was performed using an oscillating saw with an inclination of 15° on the sagittal plane and perpendicular to the second metatarsal (Figure 1B,D). After that, a 2 mm Kirschner wire was inserted at the same distance from both the superior and the inferior margin of the metatarsal bone in an antegrade fashion into the soft tissues adjacent to the bone. Slight plantarflexion of the head of the first metatarsal could be taken into consideration to restore the impaired function of the I ray. The wire emerged from the tip of the toe, just next to the medial edge of the nail. Before the K-wire insertion, the metatarsal head could be moved in the transverse plane and rotated to correct the distal metatarsal articular angle (DMAA). The wire was withdrawn from the tip of the toe until its proximal end reached the osteotomy line. Then, using a small-grooved lever to access the osteotomy, the Kirschner wire was inserted in a retrograde fashion through the osteotomy site into the diaphyseal channel of the first metatarsal bone up to the base of the metatarsal (Figure 1C,E). The proximal fragment of the osteotomy was usually medially prominent and required trimming with a Luer or an oscillating saw [5].

All patients were discharged one day after surgery. Ambulation was allowed immediately using postoperative shoes that transferred weight bearing to the hindfoot. After about 30 days, the dressing, the suture, and the Kirschner wire were removed, and radiological control was performed. Progressive rehabilitation with passive and active exercises were advised, comfortable shoes were prescribed on day 45, and the gradual recovery of normal walking was encouraged.

### 2.3. Clinical and Radiological Assessments

All patients were administered the American Orthopaedic Foot and Ankle Society’s (AOFAS) questionnaires using the Hallux Metatarsophalangeal–Interphalangeal Scale [20], which was independently completed by patients during hospitalization before surgery and at the 12-month follow-up visits. Clinical outcomes are graded for pain (from 0 to 40 points); functional evaluation (from 0 to 45 points), concerning activity limitations, footwear requirements, metatarsophalangeal and interphalangeal joints motion and stability, and calli formation; and hallux alignment (from 0 to 15 points). Thus, the overall score can range from 0 to 100 points, where 100 is a foot without pain with the hallux well aligned and no functional issue. Preoperative scores were compared with ones collected at the last follow-up. The results were then categorized as excellent (91–100 points), good (81–90 points), fair (71–80 points), and poor (70 points or less).

Moreover, pain was quantified using the Numerical Rating Scale (NRS), ranging from 0 points, which represents no pain, to 10 points, representing maximal imaginable pain [21].

The dorsoplantar and lateral weight bearing radiographs of the feet, before surgery and one, three, six, and 12 months after surgery, were assessed. We measured the HVA and IMA before surgery and at the last follow-up. The HVA is the angle between the longitudinal axes of the first metatarsal and the proximal phalanx through the antero-posterior, and the IMA is the angle between the longitudinal axes of the first and second metatarsals [22]. The radiographic evaluation and angular parameters calculation were performed by 2 independent observers, who were blinded to the clinical outcomes and reports from the radiologist.

Complications, such as non-union, delayed union, mal-union, recurrence, and iatrogenic hallux varus, were evaluated through both radiological and clinical follow-up, and were recorded when they occurred.

### 2.4. PP Measurement

**Figure 2:**
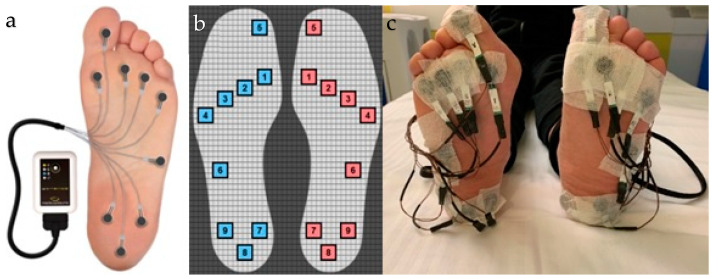
Schematic illustration of the W-INSHOE© (Medicapteurs, Balma, France) device unit connected to the 9 ultra-thin calibrated resistive sensors (**a**); the sole was divided into 9 regions and each sensor was positioned as follows: sensor number 1 corresponds to the first metatarsal head, sensor 2 to the second metatarsal head, sensor 3 to the third metatarsal head, sensor 4 to the region of the fourth and fifth metatarsal heads, sensor 5 to the hallux distal phalanx, sensor 6 to the lateral area of the midfoot, sensor 7 to the medial edge of the hindfoot, sensor 8 to the calcaneal tuberosity, and sensor 9 to the outer edge of the heel (**b**); example of sensors positioned on the patient (**c**).

All patients underwent a PP measurement before and after surgery. The PP measurement system we used in the study was W-INSHOE© (Medicapteurs, Balma, France), which is an in-shoe pressure analysis device that was previously validated and is widely used; this enabled real-time assessment of foot pressures, either in static or dynamic conditions [23,24,25,26,27]. This product consists of two ultra-lightweight units (50 g), which we applied to the patients’ ankles using Velcro tape, and each unit controlling 9 ultra-thin calibrated resistive sensors, which were positioned at specific points of the sole foot using adhesive patches applied manually by the operator. Each specific point, shown in Figure 2, corresponded to a specific region, thus dividing the sole into 9 regions. While most systems use insoles with fixed sensor locations, the W-INSHOE system is equipped with nine resistive pressure sensors that can be positioned more accurately on the anatomical area by expert practitioners (orthopedic and podiatrist). We chose this system for a more precise investigation of the specific anatomical landmarks and to exclude bias, such as the different feet sizes and the different forefoot morphotypes, such as index-plus, index plus-minus, and index-minus, that affect the systems based on insoles that are entirely wrapped in a matrix of sensors and not customized based on different foot morphotypes [28].

After equipping the patient and configuring the software, the patient was asked to walk barefoot (in order to exclude the influence of different shoes) across a flat floor at their normal pace along a three-meter corridor. The recording lasted 60 s for each analysis. The PP was measured at 50 Hz for 5 s (an average of 3.3 consecutive steps [3–4 steps]), and the analysis was performed using the W-INSHOE software.

When the units are activated, they automatically synchronize via Bluetooth with the computer interface, which allows for the real-time 3D acquisition and display of PP values. Moreover, after recording, the software allows for a detailed acquisition analysis: it can display the pressures, activation times, and each of the sensors’ impulses, and compares the right and left feet and the sensor values between several recordings.

The software recorded various parameters, including the peak pressure (peak-P), which was utilized as a comparative measure in our study.

### 2.5. Statistical Analysis

The mean values of the PP distribution for each region before surgery were compared with the values at the 12-month follow-up visit. Additionally, clinical and radiographic comparisons in the pre- and postoperative groups, as well as comparison of the PP measurements with clinical and radiological outcomes, were performed.

Categorical variables were assessed using chi-square tests, while the normality of the continuous variables was examined using the Shapiro–Wilk normality test. For variables with a normal distribution, the results were analyzed using Student’s *t*-test; conversely, the McNemar test was employed for non-normally distributed variables.

Statistical significance was set at a P-value less than 0.05, per the standard convention.

## 3. Results

### 3.1. Population

At the beginning of the study, we enrolled 18 patients, but 3 patients were lost at the PP measurement follow-up visit. The patients’ characteristics are reported in Table 1.

### 3.2. Clinical and Radiological Results

The AOFAS outcomes using the Hallux Metatarsophalangeal–Interphalangeal Scale rating [20], the NRS for pain assessment, and the main angular parameters—HVA and IMA—are reported in Table 2.

At the 12-month follow-up, the mean AOFAS scores had improved significantly from a preoperative mean of 46.7 ± 15.6 (range 28–70) to a postoperative mean of 84.8 ± 15.2 (range 45–100) points; specifically, it was excellent in five cases, good in four, fair in four, and poor in one case.

The NRS was 6.4 ± 1.3 (range 4–8) preoperatively and dropped to 2.2 ± 2.4 (range 0–6) postoperatively.

The mean preoperative HVA was 34.1 ± 7.2 degrees (range 22.2–53.7), and the mean postoperative HVA was 11.4 ± 3.8 degrees (range 6.2–23.2). Similarly, the mean preoperative IMA was 12.9 ± 2.2 degrees (range 9.4–18.2), while the mean postoperative IMA was 5.6 ± 2.2 degrees (range 2.6–9.6).

No complications were reported at the 12-month follow up.

### 3.3. Differences in Plantar Pressure Parameters before and after HV Surgery

**Figure 3:**
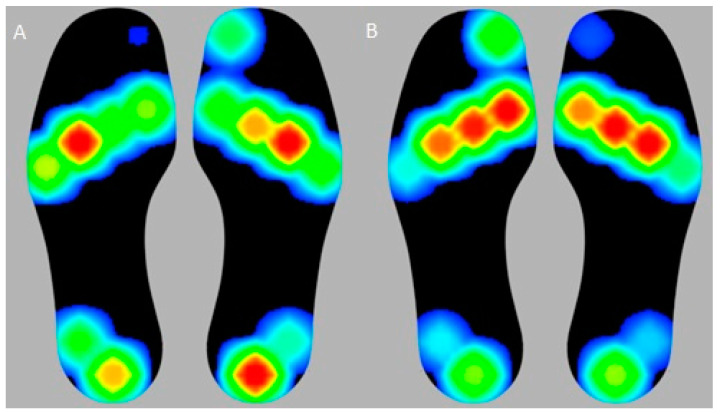
Plantar pressure distribution with color codification (pressure intensity increase from green to red) of one patient who underwent bilateral surgery, comparing pre- (**A**) and postoperative (**B**) measurements.

Generally, after surgery, a more uniform PP distribution was observed throughout the feet (Figure 3).

The first metatarsal head recorded a significant increase in the peak pressure, which was associated with a concomitant increase in the peak pressure under the central metatarsal area after surgery compared with the preoperative values (Table 3).

## 4. Discussion

Plantar pressure distribution measurement systems have recently been introduced to objectify functional outcomes after surgery. This technology is easy to use, straightforward, and may be used as a standard measurement tool to increase the results’ reliability and comparability. The purpose of the present study was to examine patients with HV before and after surgery using a plantar pressure distribution measurement system.

To our knowledge, the present study was the first to apply a plantar pressure measurement system that takes in account the anatomic asymmetry and difference in both feet, adapting the layout to each foot before and after surgery.

A S.E.R.I osteotomy is a well-established distal metatarsal osteotomy for bunion correction that has reported satisfactory clinical and radiographical outcomes over the years [5,29,30,31,32,33]. Consistently, in this study, both clinical and radiographical outcomes showed significative improvement.

W-INSHOE© (Medicapteurs, France) was used to assess the plantar pressure distribution: the device is a flexible, portable, in-sole system that enables the real-time assessment of foot pressures, either in static or dynamic conditions. Each sensor can be applied on specific points of the foot sole. Despite these features, W-INSHOE© has low spatial resolution of the data compared with platform systems due to having fewer sensors [34].

The evaluation of the hallux function in the context of foot biomechanics is a complex endeavor. To enhance the reader comprehension, this study focused solely on peak-P as a parameter for the PP analysis. This deliberate simplification was employed to provide a more intuitive and accessible perspective of the intricate biomechanics of the foot. Moreover, peak-P is the most represented parameter, which allowed for a simple comparison with the other studies in the literature [10,34,35].

Although several studies were conducted on the plantar pressure distribution in relation to HV surgery, poor evidence regarding the rebalancing of plantar pressure distribution after surgery is reported by the current literature [36].

Theoretically, successful surgery should result in a reversal of the off-loading mechanism of the first ray, which is displayed as an increase in peak-P and should indirectly lead to a simultaneous decrease in the overload on the central metatarsal head [37]. The optimal surgical treatment of HV is still under debate. In the literature, several surgical treatments have been proposed (such as the Bosch technique, MIS Chevron–Akin, and Reverdin–Isham). Complications following HV surgery will have an expected incidence of between 10% and 55% of cases [38]. The management of suboptimal results, such as transfer metatarsalgia, following the surgical treatment of HV deformity should consider the pathomechanics of the foot. Conservative measures, such as foot orthoses, may be helpful to control abnormal pronation and distribute the force away from the head of the metatarsal bone, but if conservative treatment fails, revision surgery may be indicated. Carlo Biz et al. [39] suggested using Maestro’s criteria to quantify the forefoot morphotypes and the metatarsal bones to be shortened. Maestro’s criteria represent a useful predictive value for the preoperative planning and show a significant positive correlation with the clinical outcomes of many postoperative scores. Despite the theory, a persistent overload of the central metatarsal area was reported by some studies in the literature, even if an increase in peak pressure under the hallux occurred [9,10]. Consistent with our study findings, we observed a notable improvement in the plantar pressure distribution under the first metatarsal head, along with an increase in the peak pressure under the central metatarsal area, when comparing the pre- and postoperative periods.

Only one study [40] reported an increase in peak pressure at the hallux level with simultaneous second metatarsal head offload. In this case, a first metatarsal proximal crescentic osteotomy associated with a lesser metatarsal proximal shortening osteotomy was performed. The satisfied outcomes could be related to the metatarsal proximal osteotomy, which could have higher corrective power [9,11]. Also, the concurrent lesser metatarsal osteotomies might explain the better pressure pattern compared with cases where surgery addressed the first metatarsal only. Theoretically, lesser metatarsal shortening osteotomies are indicated when the metatarsal formula is altered with relatively longer second or third metatarsals, which is responsible for an unbalanced loading pressure under the central forefoot [40,41]. However, no other study compared the lesser metatarsal shortening osteotomy with PP measurements, and it is difficult to determine a direct relationship of a specific therapeutic effect when multiple procedures were performed.

Finally, our case series and the current literature appear to agree on the correlation between the postoperative increase in peak pressure under the hallux and the satisfactory clinical and radiographic outcomes. However, the increased peak pressure under the central metatarsal area, regardless of the good clinical and radiographic results, remains challenging to interpret. A recent review [36] showed no clear relationships between clinical and radiographic results and changes in foot plantar pressure patterns. It can be hypothesized that plantar pressure measurement is a more sensitive method for assessing surgical efficacy, potentially predicting any recurrence and residual pain in the long term, or it could be a method influenced by the follow-up time, whereby the expectation is a progressive reduction in the pressure distribution under the central metatarsal area. Exploring these scenarios and others would be of interest in further studies.

This study had some limitations. The small patient sample size limited the power of the statistical analysis, although strict patient selection was performed to exclude all conditions that affect gait and plantar pressure analysis in order to avoid possible confounding factors. The heterogeneity of the PP tools used, surgical approaches, patient comorbidities, and clinical and radiographic results in the literature made the sample size calculation difficult to perform and the results were difficult to compare with the literature given the lack of studies that assess the effectiveness of HV surgery by PP measurements. However, pilot studies are important in order to avoid significant errors before implementing large-scale studies and to assess the feasibility and obtain preliminary data that can be used to design a statistically adequate large-scale study. Regarding loss to follow-up, it should be noted that the lost cases included patients who were evaluated and had reported satisfactory clinical and radiographic outcomes but declined to continue participating in the ongoing experimental study. Therefore, while the loss of three patients at follow-up may pose a challenge in interpreting the results of a study with a small sample size, there are circumstances in which this loss may not be statistically significant, as in the present case, when the results were preliminary or the lost patients did not exhibit unique characteristics compared with the other participants. Although the mean follow-up duration was limited to 12 months, this time frame was deemed adequate by previous studies to achieve a stable rebalancing of plantar pressure following surgery [36]. A major limitation at present is the lack of standardization of these tools, particularly with regard to data extraction and the subsequent comparison between scientific studies. While improvements or deteriorations from baseline can be demonstrated, their absolute magnitude is difficult to quantify.

However, this study showed the simplicity and utility of this innovative tool. Incorporating a PP measurement system into routine assessments for patients has the potential to enhance our understanding of the pathology and effectiveness of surgery, ultimately contributing to more comprehensive and insightful patient care. To date, these findings warrant further scientific investigation to assess all the factors that may influence the results in terms of plantar pressure distribution, particularly the impact of the first metatarsal head plantar dislocation after the distal osteotomy, the influence of the metatarsal formula, the stiffness of the first metatarsophalangeal joint, the role of the rearfoot, and the contribution of the BMI. In addition, conducting evaluations at different time points at a longer follow-up and with different surgical approaches would provide valuable insights.

## 5. Conclusions

The main contribution of this study was to achieve greater insight into the efficacy of HV surgery (distal metatarsal osteotomy with the S.E.R.I. technique) through an innovative PP technology that was useful to objectify the postoperative hallux functional outcome. While improvements at the level of the hallux was observed, a concurrent rebalancing of the central forefoot area was not consistently reported. Nevertheless, the discrepancies in plantar pressure findings did not appear to affect the clinical and radiographic outcomes. The PP measurement system seems to be a promising additional tool in clinical practice; future research should focus on standardizing the measurement setup and selecting the most informative parameters.

## Figures and Tables

**Table 1:** Patient’s characteristics.

Patients	15 (24 Halluces)
Side	2 left, 4 right, 9 bilateral
Age	53.9 ± 17.3 (18–78)
Gender	14 F/1 M
BMI	24.1 ± 3.2

**Table 2:** Clinical and radiographic outcomes.

	Baseline	Follow-Up (12 Months)	*p*-Value
AOFAS	46.7 ± 15.6	84.8 ± 15.2	<0.001 *
Pain	20.8 ± 10.8	36.7 ± 6.5	<0.001 *
Function	21.4 ± 8.8	34.3 ± 8.7	<0.001 *
Alignment	4.5 ± 6.0	13.8 ± 2.7	<0.001 *
NRS	6.4 ± 1.3	2.2 ± 2.4	<0.001 *
IMA	12.9° ± 2.2	5.6° ± 2.2	<0.001 *
HVA	34.1° ± 7.2	11.4° ± 3.8	<0.001 *

*Abbreviations*: **AOFAS**—American Orthopaedic Foot and Ankle Society; **NRS**—Numerical Rating Scale; **IMA**—intermetatarsal angle; **HVA**—hallux valgus angle. * **Bold** *p*-value indicates reaching statistical significance.

**Table 3:** Peak pressure (peak-P) measurement outcomes.

Peak-P	Baseline	Follow-Up (12 Months)	*p*-Value
Region 1	773.1 ± 663.5 (66.2–2148.2)	1088.8 ± 236.7 (582.7–1545.5)	0.027 *
Region 2	552.2 ± 256.7 (139.4–1290.5)	844.1 ± 317.6 (260.8–1424.5)	0.005 *
Region 3	714.71 ± 366.6 (118.5–1483.2)	999.6 ± 383.1 (345.6–1858.1)	0.024 *
Region 4	515.9 ± 402.8 (67.0–1586.1)	631.7 ± 304.8 (31.7–1267.9)	0.266
Region 5	264.8 ± 316.0 (35.6–1419.3)	415.7 ± 324.5 (0.5–1153.4)	0.107
Region 6	134.7 ± 166.5 (14.4–779.9)	162.4 ± 128.8 (37.2–479.9)	0.474
Region 7	116.3 ± 195.7 (0.7–732.4)	78.0 ± 121.8 (0.4–454.4)	0.472
Region 8	516.7 ± 274.3 (145.4–1293.2)	528.9 ± 305.9 (13.0–1136.8)	0.896
Region 9	269.7 ± 280.9 (1.2–1158.2)	228.6 ± 212.6 (1.2–926.5)	0.394

Region 1: first metatarsal head, region 2: second metatarsal head, region 3: third metatarsal head, region 4: fourth and fifth metatarsal heads; region 5: hallux distal phalanx, region 6: lateral area of the midfoot, region 7: medial edge of the hindfoot, region 8: calcaneal tuberosity, region 9: outer edge of the heel. The measurements of peak pressure are reported in kPa. * **Bold** *p*-value indicates reaching statistical significance.

## Data Availability

The data that support the findings of this study are available from the corresponding author, but restrictions apply to the availability of these data, which were used under license for the current study, and thus, are not publicly available. Data are, however, available from the authors upon reasonable request and with the permission of the corresponding author.
